# Falls and falls efficacy: the role of sustained attention in older adults

**DOI:** 10.1186/1471-2318-11-85

**Published:** 2011-12-19

**Authors:** Aisling M O'Halloran, Nils Pénard, Alessandra Galli, Chie Wei Fan, Ian H Robertson, Rose Anne Kenny

**Affiliations:** 1 TRIL (Technology Research for Independent Living) Centre, St. James's Hospital, Dublin, Ireland; 2 TRIL (Technology Research for Independent Living) Centre, Trinity College, Dublin, Ireland; 3 Trinity College Institute of Neuroscience, Trinity College, Dublin, Ireland; 4 Mercer's Institute for Successful Ageing, St. James's Hospital, Dublin, Ireland; 5 Department of Medical Gerontology, Trinity College, Dublin, Ireland

## Abstract

**Background:**

Previous evidence indicates that older people allocate more of their attentional resources toward their gait and that the attention-related changes that occur during aging increase the risk of falls. The aim of this study was to investigate whether performance and variability in sustained attention is associated with falls and falls efficacy in older adults.

**Methods:**

458 community-dwelling adults aged ≥ 60 years underwent a comprehensive geriatric assessment. Mean and variability of reaction time (RT), commission errors and omission errors were recorded during a fixed version of the Sustained Attention to Response Task (SART). RT variability was decomposed using the Fast Fourier Transform (FFT) procedure, to help characterise variability associated with the arousal and vigilance aspects of sustained attention.

The number of self-reported falls in the previous twelve months, and falls efficacy (Modified Falls Efficacy Scale) were also recorded.

**Results:**

Significant increases in the mean and variability of reaction time on the SART were significantly associated with both falls (p < 0.01) and reduced falls efficacy (p < 0.05) in older adults. An increase in omission errors was also associated with falls (p < 0.01) and reduced falls efficacy (p < 0.05). Upon controlling for age and gender affects, logistic regression modelling revealed that increasing variability associated with the vigilance (top-down) aspect of sustained attention was a retrospective predictor of falling (p < 0.01, OR = 1.14, 95% CI: 1.03 - 1.26) in the previous year and was weakly correlated with reduced falls efficacy in non-fallers (p = 0.07).

**Conclusions:**

Greater variability in sustained attention is strongly correlated with retrospective falls and to a lesser degree with reduced falls efficacy. This cognitive measure may provide a novel and valuable biomarker for falls in older adults, potentially allowing for early detection and the implementation of preventative intervention strategies.

## Background

One-third of people over the age of 65 have at least one fall each year and almost half of these experience more than one fall [1,2]. Falls are a major cost to healthcare systems worldwide and have significant adverse impacts both physically and psychologically on the older person. Following a fall, older people often voluntarily restrict their activity fearing a reoccurrence. This reduction in exercise leads to further weakness that in turn increases the risk of another fall -- a vicious cycle [3]. Currently, intervention strategies targeted to known risk factors only result in a 30-40% reduction in the reoccurrence of falls after one year [4-6]. This highlights the need to identify additional risk factors which contribute to falls and provide novel interventions to lower falls risk more effectively.

Low falls-related self efficacy (loss of one's confidence to perform activities of daily living without falling) and fear of falling are significant psychological consequences of having fallen [7]. While low falls efficacy and fear of falling were traditionally considered interchangeable concepts, more recent evidence indicates that they are correlated but distinct dimensions [3,8,9]. Both low falls efficacy and fear of falling are associated with previous and future falls [10-15], however falls efficacy is a stronger predictor of falls and can be considered a mediator between fear of falling and falls [15]. Interestingly, fear of falling has been reported in older people who have not fallen suggesting that factors other than falls history may influence the manifestation of fear of falling among older people [12,16].

Several studies have suggested that aspects of cognition, particularly declining executive function, are correlated with and predictive of falls risk in older adults without dementia or overt cognitive impairment [17-19]. Since deficits in executive function increase with age, this may impair the ability of the older person to compensate for age-related changes in gait and balance. This in turn, may compromise the ability of the older person to negotiate and cope with the complexities of their day to day surroundings [20-22]. This is supported by evidence from gait and balance studies, particularly dual tasks, indicating that gait performance and falls are related to executive function, and that falls have been associated with primary ageing of the prefrontal cortex [23,24]. Attention is a specific component of executive functioning. Low scores on tests of attention have been correlated with postural instability and increasing gait variability, both of which are related to falls [25,26]. Additional findings revealed that older people allocate more of their attentional resources toward their gait and that the attention-related changes that occur during aging increase the risk of falls [27,28].

Sustained attention is fundamental executive function in attaining complex goals that require monitoring, over time [29]. Successful sustained attention performance is due to the workings of two interacting subsystems: vigilance and arousal [30]. Vigilant attention is a top-down system that relies on a right lateralized network of cortical areas including the cingulate gyrus, prefrontal cortex and inferior parietal lobule [31,32]. Arousal is a bottom-up, subcortical system mediated through a subcortical network including the thalamus and noradrenergic brainstem structures, including the locus coeruleus [33,34]. Sustained attention is modulated by noradrenergic activation. Noradrenaline (NA) is produced by the locus coeruleus, and has a widespread distribution to cortical areas of the brain including the right lateralised fronto-parietal region [35,36].

In this study, we measured attentional performance and variability in older fallers and non-fallers, without cognitive impairment, using the Sustained Attention to Response Task (SART) [37]. Imaging studies of this task confirmed robust activation within right-lateralised fronto-parietal and subcortical attentional networks [31,32]. The SART provides ample time-series, reaction time (RT) data for analysis using the Fast Fourier Transform (FFT) procedure which distinguishes discrete components of RT variability. Slow variability, which has a slow temporal characteristic, can be differentiated from fast variability, which has a fast moment-to-moment temporal characteristic. Recent studies by Johnson *et al *suggest that changes in slow variability may reflect alterations in arousal, while changes in fast variability may reflect fluctuations in the control of vigilant attention [38-40]. We hypothesised that increasing RT variability and higher error rates, signifying poorer sustained attention, would be associated with falls and falls efficacy due to a combination of age-related NA depletion and primary ageing of the frontal cortex. Such age-related changes lead to a decline in top-down functioning and processing speed [41,42]. Therefore, we also anticipated that more variability in the fast variability (vigilance) aspect of sustained attention would be particularly associated with falls.

## Methods

### Setting

A convenience sample of 624 men and women aged ≥ 60 years underwent a comprehensive multidisciplinary geriatric assessment at the Technology Research for Independent Living (TRIL) Clinic, St James's Hospital, Dublin, Ireland, between August 2007 and May 2009. The majority of participants (66.8%) were self-referrals for ''health check'' attracted by the TRIL Centre website http://www.trilcentre.org or articles in the local media. The remainder (33.2%) were referred from medical and health professionals for further assessment of participants with a history of falls. Participants did not have familiarization visits, but prior to attendance they had a telephone conversation with the clinical nurse specialist explaining the content of the scheduled visit.

### Participants

All 624 participants were ≥ 60 years old, community-dwelling, medically stable, without a history of stroke, or dementia (MMSE score ≥23), able to walk independently (with or without aids), and able to provide written informed consent. Participants were not asked to stop any of their usual medications, fast, or modify lifestyle habits before assessment. Of these, 500 individuals were offered additional cognitive tests including the Sustained Attention to Response Task SART [37], of which 458 successfully completed this task. The 42 participants who did not complete the task did so due to fatigue or a lack of understanding of the task instructions. The group of 166 individuals who were either not offered or did not complete the SART were significantly older (77.2 versus 71.6 years), with lower mean MMSE scores (25.8 versus 27.8), and had a higher percentage of fallers (65.6% versus 43.3%) compared to those who completed the SART.

### Physical, psychosocial and cognitive measurements

Physical measurements included age, gender, weight (kg) and height (cm). The timed up and go test (TUG): measured the length of time (seconds) taken to get up from a sitting position, walk 3 metres, turn around, walk back and resume a sitting position. It is a widely used measure of gait and balance in older people, with longer TUG times (> 11 seconds) associated with a higher risk for falls [43]. The Berg balance scale (BBS): a 14-item scale that measured balance among older people by assessing their performance on functional tasks. Higher scores indicated better balance with minimum and maximum scores of 0 to 56 [44]. The activities of daily living scale (ADL): an 8-item scale that measured the ability to perform daily self-care activities e.g. self-washing, dressing and feeding, as measure of functional status and/or disability. Higher scores indicated better functional activity with minimum and maximum scores of 0 to 24 [45]. The instrumental activities of daily living scale (IADL): a 9-item version of the scale was used which is not a measure of fundamental functioning, but of an individual's ability to live independently in a community. Higher scores signify greater independence with minimum and maximum scores of 0 to 27 [46]. Polypharmacy was determined by the use of ≥ 4 medications daily. The Charlson co-morbidity Index (CCI) was used to classify and score the number of comorbid conditions and is a measure of disease burden. Higher scores indicate greater comorbidity and increased mortality risk with a range in this study sample of 0 - 12 [47].

Psychosocial measures included the anxiety section of the Hospital Anxiety and Depression Scale (HADS), which is a 7-item scale with higher scores reflective of greater levels of anxiety and with minimum and maximum scores of 0 to 21 [48]. A simplified 8-item version of the Center for Epidemiologic Studies Depression scale (CES-D) was used to measure depression. Higher scores reflected increasing levels of depression with minimum and maximum scores of 0 to 8 [49].

Measures of cognitive function included the mini-mental state examination (MMSE) as a measure of global cognition with higher scores to a maximum of 30 indicating better levels of cognition [50]. The cognitive failures questionnaire (CFQ) is a 25-item scale that measured self-reported absentmindedness or everyday lapses in perception, memory, and motor function. Higher scores reflect increasing levels of absentmindedness or cognitive lapses, with minimum and maximum scores of 0 to 100 [51].

### Ethics

Ethical approval was obtained from the St. James's Hospital/Adelaide and Meath Hospital, incorporating the National Children's Hospital Research Ethics Committee (approval reference number 2007/06/13). All participants gave written informed consent before inclusion in the study.

### Sustained Attention to Response Task (SART)

#### Apparatus and procedure

The SART is a computerized task and participants were shown a repeated series of fixed digits from 1 to 9 on the screen. Participants were instructed to "press a key for every number that you see as fast as you can (go answer) but try not to press on the number 3 (no-go answer)". Each digit appeared for 300 milliseconds (ms), with an interval of 800 ms before the next digit appeared. The cycle of digits 1 to 9 was repeated 23 times, giving a total of 207 trials. During a pilot, we noticed that some participants had difficulties performing the SART for the whole length of the task. In order to minimize data attrition, we reduced the number of trials from 225 in the original SART to 207 trials. The task lasted approximately 4 minutes. The recorded variables were: mean reaction time (mean RT) i.e. the time taken for the key presses on digits 1, 2 and 4 to 9 across the entire task; standard deviation (variability) of reaction time (SDRT), the number of commission errors i.e. inappropriate key presses in response to the digit 3, and the number of omission errors i.e. failure to press a key in response to digits 1, 2 and 4 to 9 [37].

#### Data pre-processing for FFT analysis

In addition to the traditional analysis of behavioural responses, we also analyzed the reaction time data of the participants using the Fast Fourier Transform (FFT) procedure described previously [38-40]. Briefly, overall variability in reaction time results from the combination of different sources of variance occurring on different time scales. For instance, the variance of a given participant can be made up of a continuous slowing down of the reaction time over the length of the task (slow frequency variability - SFV), and the quick changes occurring on a moment to moment basis (fast frequency variability - FFV). In this paper, we use the FFT procedure to decompose the variance of the reaction times into these two additive components. The FFT procedure can only be applied to continuous, non-zero data, and so pre-processing of the SART reaction time data prior to the FFT analysis was necessary. Therefore, we interpolated zero values (corresponding to omission errors and correct no-go responses on digit 3) using the two reaction times before and after the zero values. Participants that exhibited gaps too large to be considered continuous for the application of the FFT procedure, could not be adequately interpolated using averaging methods. In this context, 74 participants with more than 6 consecutive zero answers were removed from the analysis reducing the number of participants with FFT data to 384. The group of 74 individuals with more than 6 consecutive zero answers were significantly older (75.4 versus 71.0 years, p < 0.001), with lower mean MMSE scores (26.6 versus 28.0, p < 0.001) but not CFQ scores, and had a higher percentage of fallers (56.6% versus 40.7%, p = 0.011) but did not differ significantly by MFES score compared to those with less than 6 consecutive zero answers. The excluded group were physically in poorer health with higher levels of comorbidity (4.9 versus 3.3, p < 0.001), poorer gait from the TUG times (11.0 versus 8.8 s, p < 0.001), and poorer Berg balance scores (49.9 versus 53.1, p < 0.001). They also had lower IADL scores (25.2 versus 26.1, p = 0.001) and reduced levels of visual contrast sensitivity (1.60 versus 1.68, p = 0.001). The two groups did not differ by depression or anxiety score. Those with more than 6 consecutive zero answers also performed significantly more poorly on the traditional SART measures with longer mean RT (466 versus 381 ms, p < 0.001), longer SDRT (227 versus 147 ms, p < 0.001), more commission errors (8.7 versus 5.2, p = 0.001) and more omission errors (42.7 versus 9.3, p < 0.001).

### History of falls

Participants who experienced one or more falls in the previous twelve months were classified as a faller (n = 197). Non-fallers had not experienced a fall in the last twelve months (n = 261). Participants were further categorised by faller type: non-faller (n = 261, no falls in previous twelve months); single faller (n = 120, one fall in previous twelve months) and recurrent faller (n = 77, two or more falls in previous twelve months) [23,52].

### Falls efficacy

Falls efficacy was measured using the Modified Falls Efficacy Scale (MFES); this was a 14-item self report scale measuring confidence in one's ability to avoid falling during the performance of activities of daily living (ADL) [7]. Subjects were asked to rate their confidence in performing each activity without falling on a 0 - 10 scale, the average score across all 14 items was taken, with a minimum score of 0 indicating no confidence and a maximum score of 10 indicating full confidence (high falls efficacy) in performing the tasks without falling (table 1).

**Table 1:** Basic characteristics of fallers and non-fallers

Participant Characteristics	Non-Fallers (n = 261)	Fallers (n = 197)	p-value
N*	261 (57.0)	197 (43.0)	-
Gender - Female*	164 (62.8)	157 (79.7)	< 0.001
Age	70.27 (6.42)	73.48 (7.33)	< 0.001
Body Mass Index (BMI)	27.27 (4.71)	26.38 (4.64)	0.046
Timed up and go (TUG)	8.23 (2.21)	10.41 (4.60)	< 0.001†
Berg Balance scale	54.02 (3.15)	50.77 (6.62)	< 0.001
Activities of Daily Living scale (ADL)	22.79 (1.66)	22.39 (1.77)	0.008
Instrumental Activities of Daily Living scale (IADL)	26.52 (1.28)	25.26 (2.78)	< 0.001
Mini mental state Examination (MMSE)	28.07 (1.70)	27.34 (2.57)	0.013
Cognitive Failures Questionnaire (CFQ)	33.52 (14.31)	36.07 (14.38)	0.057
Modified Falls Efficacy Scale (MFES)	9.68 (0.72)	8.77 (1.68)	< 0.001
Center of Epidemiological Study Depression Scale-8 item (CESD8)	1.35 (1.69)	1.92 (1.96)	0.001
Hospital Anxiety and Depression Scale (HADS)	4.74 (2.88)	5.79 (3.82)	0.010
Charlson Comorbidity Index (CCI)	1.33 (1.63)	2.18 (2.08)	< 0.001
Polypharmacy (≥4 medications daily)*	100 (38.3)	115 (58.4)	< 0.001

Numbers represent means (standard deviations) and numbers with asterisks (*) represent counts (percentages). P-values indicate differences between fallers and non-fallers from independent *t *tests for continuous parametric variables, Mann Whitney *U *tests for non-parametric scale variables and χ^2 ^tests for categorical variables.

### Statistical analysis

Statistical analyses were performed using SPSS 16.0 statistical software packages. Differences between non-fallers and fallers were measured using independent samples *t*-tests for continuous, normally distributed variables, Mann Whitney (*U*) tests for non-parametric scale variables, and Pearson χ statistics for categorical variables. *One-way ANOVA *with *post hoc *Bonferroni pairwise testing was utilised to analyse variance differences in SART measures across the three faller types.

Multivariate logistic regression analyses with non-faller versus faller as the binary dependent variable were performed. Each SART variable was included as an independent variable in separate regression models along with age and gender to allow for the contribution of these factors. For independent variables which remained in the model as significant factors associated with falling, p-values, odds ratios (OR) with 95% confidence intervals (CI) were provided.

MFES scores did not conform to a normal distribution with 70% of participants scoring 9.0 or more on the 0-10 scale. This resulted in skewing of the MFES scores. A statistical measure of skewness (G_1_) = -2.43, which is larger than twice its standard error (SE) = +/- 0.10, confirmed that the distribution of the MFES scores differed significantly from a normal distribution. For this reason non-parametric tests were employed including Mann Whitney (*U*) tests to compare MFES scores between non-fallers and fallers or males and females. Similarly, Spearman *rho *(r_s_) correlations were computed between MFES score and the SART measures. Partial correlations between MFES score and the SART measures with age and gender partialled out were also performed.

## Results

### Fallers versus non-fallers

**Figure 1:**
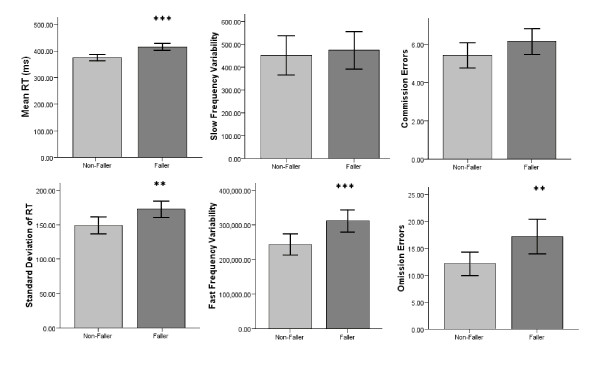
**Comparison of SART measures (means) between non-fallers and fallers**. Mean reaction time (mean RT) in milliseconds (ms), standard deviation of reaction time (Standard Deviation of RT), slow frequency variability (SFV), fast frequency variability (FFV), mean commission errors and mean omission errors were compared between non-fallers and fallers in the previous twelve months. Error Bars represent of 95% confidence intervals (CI). Significant differences are indicated at the level: *p < 0.05, **p < 0.01 and ***p < 0.001.

Differences in the physical, psychosocial and cognitive measurements between non-fallers and fallers are summarised in Table 1 and a comparison of the SART measures between non-fallers and fallers is shown in Figure 1. There was a significant difference in mean RT (*t(456) *= 4.40, p < 0.001), SDRT (*t(456) *= 2.74, p = 0.006), FFV (*t *(382) = 3.10, p = 0.002) and omission errors (*t(456) *= 2.55, p = 0.011) between fallers and non-fallers. However, there were no significant differences in SLV or commission errors.

Even when the group which were excluded from the FFT analysis (n = 74) were also removed for the analysis of the traditional SART measures significant differences remained between fallers (n = 157) and non-fallers (n = 227) for mean RT (396 versus 370 ms, p = 0.007), SDRT (161 versus 138, p = 0.009) and omission errors (10.5 versus 8.5, p < 0.014) but not commission errors.

There were significantly more female fallers compared to male fallers (58.4% versus 34.6% respectively; χ^2^(1) = 21.73, p < 0.001). Also, fallers were significantly older (*t(458) *= 7.26, p < 0.001) compared to non-fallers as can be seen from Table 1. The results from binary logistic regression models between non-fallers and fallers including each of the SART variables adjusting for age and gender are shown in Table 2. Only mean RT (p = 0.038, OR = 1.00, 95% CI: 1.00 - 1.01) and FFV (p = 0.009, OR = 1.14, 95% CI: 1.03 - 1.26) were significant factors associated with falls in the past year along with age and gender.

**Table 2:** Logistic regression modelling of SART measures between non-fallers and fallers controlling for age and gender

Dependent Variable: non-faller versus faller					
**Logistic Model**	**Independent Variables**	**Factor Coefficients**			
		
		**p-value**	**OR**	**Lower** **95% CI**	**Upper** **95% CI**

1	Mean Reaction Time	0.038	1.00*	1.00	1.01
	Age	< 0.001	1.06	1.03	1.10
	Gender	< 0.001	0.44	0.28	0.68
2	Standard Deviation of Reaction Time	0.174	1.00	0.99	1.00
	Age	< 0.001	1.07	1.03	1.10
	Gender	< 0.001	0.43	0.28	0.97
3	Slow Frequency Variability from FFT	0.943	1.00	0.97	1.04
	Age	< 0.001	1.06	1.03	1.10
	Gender	< 0.001	0.42	0.26	0.68
4	Fast Frequency Variability from FFT	0.009	1.14*	1.03	1.26
	Age	0.001	1.06	1.02	1.09
	Gender	0.001	0.43	0.26	0.69
5	Commission Errors	0.825	1.00	0.96	1.04
	Age	< 0.001	1.07	1.04	1.11
	Gender	< 0.001	0.42	0.27	0.65
6	Omission Errors	0.126	1.01	0.99	1.02
	Age	< 0.001	1.07	1.04	1.10
	Gender	< 0.001	0.43	0.27	0.67

Significant independent variables in the models are indicated with p-values and odds ratios (OR) with 95% confidence intervals (CI). Asterisks (*) indicate SART variables significantly associated with fallers.

### Faller type

**Figure 2:**
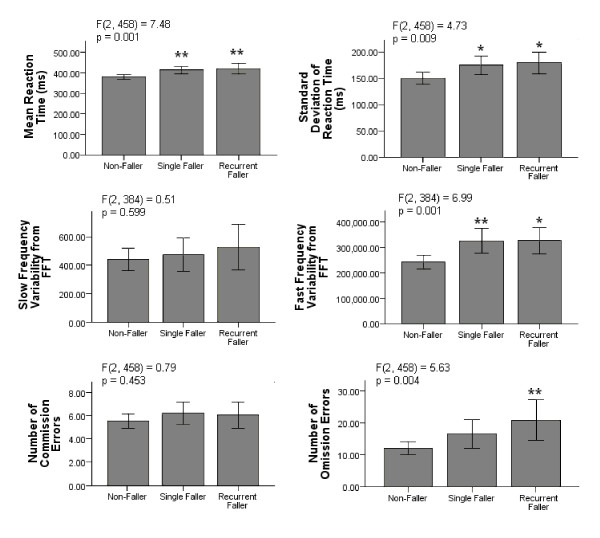
**Summary of SART measures (means) and faller type**. Mean reaction time (mean RT) in milliseconds (ms), standard deviation of reaction time (Standard Deviation of RT), slow frequency variability (SFV), fast frequency variability (FFV), mean commission errors and mean omission errors were compared between non-fallers, single fallers and recurrent fallers in the previous twelve months. Error Bars represent 95% confidence intervals (CI). Significance differences between non-fallers versus single fallers and non-fallers versus recurrent fallers are indicated at the level: *p < 0.05 and **p < 0.01.

A comparison of the SART measures between non-fallers, single fallers and recurrent fallers is shown in Figure 2. One-way ANOVA testing revealed that mean RT differed significantly with faller type (*F(2,458) *= 7.48, p = 0.001). Mean RT increased for both single (p = 0.008) and recurrent fallers (p = 0.006), compared to non-fallers. A similar pattern was seen with respect to SDRT (*F(2,458) *= 4.73, p = 0.009). Once again variability was greater for single (p = 0.046) and recurrent fallers (p = 0.042), compared to non-fallers. The FFV measure of variability also differed significantly with faller type (*F(2,384) *= 6.99, p = 0.001). This time the greatest difference was between non-fallers and single fallers (p = 0.004) although there was also a significant difference between non-fallers and recurrent fallers (p = 0.024). Finally there was a global association between faller type and omission errors (*F(2,458) *= 5.63, p = 0.004), this association was accounted for by the difference between recurrent fallers and non-fallers (p = 0.005). There were no statistical differences in SLV or commission errors.

### Falls efficacy

MFES scores were negatively correlated with mean RT, SDRT, FFV and omission errors in the faller group (Correlation range: r_s_(197) = -0.173 to -0.195, p < 0.05). There were no significant correlations between MFES score and SLV or commission errors among fallers. Meanwhile in non-fallers MFES scores were negatively correlated with mean RT, SDRT, FFV, SLV and omission errors (Correlation range: r_s_(261) = -0.137 to -0.203, p < 0.05). Among non-fallers MFES score was not significantly correlated with commission errors

Fallers had lower mean MFES scores compared to non-fallers (8.85 versus 9.56, p < 0.001) and females had lower MFES scores compared to males, (p < 0.001). MFES score was also significantly correlated with age (r_s_(458) = -0.300, p < 0.001). Partial correlations were performed between MFES score and the SART measures in fallers and non-fallers, with age and gender partialled out. Only mean RT (r(261) = -0.159, p = 0.012) and FFV (r(218) = -0.122, p = 0.073) remained weakly correlated with MFES score and only in the non-faller group.

## Discussion

In this study we explored the relationships between sustained attention, falls, and falls efficacy. We found that longer mean reaction times and more variability of reaction time along with higher numbers of omission errors during the sustained attention to response task (SART) was significantly associated with a history of at least one fall in the previous 12 months. Logistic regression modelling of the SART measures controlling for age and gender, revealed that greater variability in reaction time (particularly fast frequency variability or FFV) was strongly associated with falls. In fact a unit increase in this variability measure increased the risk of a previous fall in the past year by 14%. These results suggest that increasing variability in the executive or vigilance aspect of sustained attention is associated with falls in older adults and may represent a novel marker of falls risk. Previous studies have reported significant associations between declining executive function and falls [17-19,23], or low scores/longer reaction times on tests of attention and increasing gait variability or postural instability, both of which are related to falls However reports looking specifically at attention have tended to focus on divided or selective attention during dual tasking or have selected older adults with cognitive impairment, dementia, stroke and Parkinson's disease (PD)., [25-28,53]. This paper directly associates reduced levels of sustained attention (and particularly vigilant attention) as measured by the SART) and falls in older community dwelling adults without a history of overt cognitive impairment, dementia, stroke or PD.

We next investigated faller type and found that mean and variability of reaction time, and omission errors, again differed markedly. The strongest differences were observed for recurrent fallers compared to non-fallers, although single fallers also differed significantly from non-fallers. In contrast, the strongest difference in the fast (moment to moment) component of variability were between single fallers and non-fallers, although significant differences between recurrent and non-fallers were also observed. These results are consistent with reports of other cognitive profiles in recurrent and single event fallers [18,23]. The lengthening of reaction times, accompanied by increasing variability in reaction time and higher omission error rates may indicate that lapsing sustained attention may already have begun to manifest itself in those who have experienced just one fall. These results also suggest that insufficiencies of sustained attention are even more pronounced in recurrent fallers because recurrent falls are more likely to be indicative of neuropathology than a single fall [18].

Falling was strongly associated with low falls efficacy, exhibited by a significantly lower MFES scores in fallers versus non-fallers. Lower MFES score was correlated with longer mean reaction time and more variability of reaction time in both fallers and non-fallers. Lower MFES score was also significantly correlated with higher numbers of omission errors (but not commission errors) in both fallers and non-fallers. Partial correlations with age and gender partialled out revealed mean reaction time and the fast variability component of sustained attention (FFV) were both weakly correlated with MFES scores but only in the non-faller group. This is difficult to interpret but perhaps here lower falls efficacy may be mirroring fear of falling, which is reported by 20-85% of community-dwelling non-fallers [54]. Fear of falling is correlated with low falls efficacy, and factors other than a previous fall can induce both outcomes in older adults. The interaction between falls and lower falls efficacy, and the influence of gender and age, is in keeping with previous studies, suggesting that sustained attention and falls efficacy may be interacting to influence falls risk [7-9,55].

It must be noted that longer mean reaction times (among fallers) during the SART may indicate more careful and focused attention to task, rather than slowed processing speed or deficits in sustained attention *per se *[56]. This view is supported by the fact that non-fallers and fallers made similar numbers of commission errors, a measure of drifting attention in the fixed version of the SART. Thus, in order to achieve similar levels of accuracy during the SART (as determined by the number of commission errors) fallers must slow their reaction times compared to non-fallers. There are two possible explanations for this. Firstly, a general slowing down over the course of the SART has been shown to improve attentional control and hence accuracy on the task [56]. Alternatively, longer mean reaction times may represent a compensatory strategy which may be characteristic of fallers compared to non-fallers. For this reason, differences in variability of reaction time on the SART are generally a more reliable marker of declining sustained attention than mean reaction time.

There is some evidence indicating that greater fast frequency variability (FFV) may reflect deficits in the vigilant attention system. This is supported by research indicating that children with ADHD exhibit increases in the fast frequency component of variability on the SART suggesting a loss of executive functional control, possibly involving the right hemispheric fronto-parietal system, which may then be responsible for deficits in sustained attention [38,39,57]. In this context, the data presented here suggest that more variability in the executive aspect of sustained attention may represent a potential marker of declining sustained attention that is associated with falling. This may be further evidence that falls are associated with deficits in top-down (fronto-parietal) systems that are necessary for cognitive control of gait and postural stability. Neuroimaging of older fallers and non-fallers performing this sustained attention test will help to clarify the exact areas of dysfunction.

This study was a convenience sample of medically stable, community-dwelling older adults who were able to walk independently (with or without aids), and may be a potential design limitation. It may be considered more representative of a relatively well sample of older adults rather than representative of the general population over 60 years which would include non-mobile, medically unwell and non-community-dwelling individuals. There were also a high percentage of females in the sample, which can be explained for two reasons. Firstly, this convenience sample was two-thirds self-referred and older females are more likely to engage in volunteered participation than older males. Secondly, one-third of the sample was medically referred with a history of falls and females are more likely to fall and require medical treatment than males. Since falls history in the past twelve months was self reported and thus we must acknowledge that it may be subject to the recall bias inherent to retrospective falls data collection, whereby falls are underreported or forgotten. However, previous studies have shown that 12-month self-reported falls history had high specificity with respect to the gold standard of prospective falls assessment, especially for a cognitively intact sample of older adults without a history of dementia, which this study sample was [23,52,58]. Finally, this was a cross-sectional study of older adults and therefore we can not yet confirm causality i.e. that variability of sustained attention is a predictor of future falls. A longitudinal follow up of this cohort is nearing completion and may help to answer this question.

Falls prevention is a major issue in promoting successful ageing and fostering independence among the older population. Current intervention strategies targeted to known risk factors only result in a 30-40% reduction in the reoccurrence of falls [4-6] This highlights the need to identify additional risk factors which contribute to falls and provide novel interventions to lower falls risk more effectively. The recent "Clinical Practice Guidelines: Prevention of Falls in Older Persons"" as set out by the American Geriatric Society (AGS) and British Geriatric Society (BGS) do not incorporate a measure or assessment of sustained attention under "Neurological function" [59]. The SART is a 4 minute test and was successfully completed by 91.6% of older adults who were offered the task. Thus, if the findings presented here for retrospective falls were replicated for prospective falls, it would confirm the potential use of this test in the clinical setting. The SART is a short, inexpensive, easily administered test of sustained attention that could provide an objective, cognitive biomarker of falls risk and reduced falls efficacy. This was also a relatively well sample of older adults indicating that the test may be capable of detecting sub-clinical differences in sustained attention allowing for early detection and intervention. In fact even when the group of poorest performers on the task (i.e. those seventy-four individuals who had six consecutive zero responses and were excluded from the FFT analysis) were removed from the analysis, significant differences in the SART measures between fallers and non-fallers remained. Many aspects of attention are amenable to interventions such as attentional training, therefore the opportunity exists to implement such strategies to improve levels of attention and decrease the risk and prevalence of falls in older adults [60,61].

## Conclusions

In conclusion we provide evidence that greater variability in sustained attention, as measured by the SART, is strongly correlated with both single and recurrent event falls in older community-dwelling adults, without cognitive impairment. In addition, greater variability in sustained attention is also weakly correlated with lower falls efficacy in older adults, particularly in those without a history of falls in the previous year. We suggest that declining sustained attention contributes to an increasing falls risk and that noradrenaline depletion and primary changes in the frontal cortex, are the age-related mechanisms responsible. Sustained attention variability may provide a novel and valuable biomarker for falls in older adults, potentially allowing for early detection and the implementation of preventative intervention strategies.

## Competing interests

The authors declare that they have no competing interests.

## Authors' contributions

Acquisition of data: NP, AG, CWF. Analysis and interpretation of data: AOH, NP. Drafting of manuscript AOH. Critical revision of the manuscript for important intellectual content: AOH, AG, CWF, IHR, RAK. Administrative, technical or material support: AG. Supervision: IHR, RAK. Final approval for publication: AOH, NP, AG, CWF, IHR, RAK.

## Pre-publication history

The pre-publication history for this paper can be accessed here:

http://www.biomedcentral.com/1471-2318/11/85/prepub
